# First Insights Into Within Host Translocation of the *Bacillus cereus* Toxin Cereulide Using a Porcine Model

**DOI:** 10.3389/fmicb.2018.02652

**Published:** 2018-11-07

**Authors:** Tobias Bauer, Wolfgang Sipos, Timo D. Stark, Tobias Käser, Christian Knecht, Rene Brunthaler, Armin Saalmüller, Thomas Hofmann, Monika Ehling-Schulz

**Affiliations:** ^1^Department of Pathobiology, Functional Microbiology, Institute of Microbiology, University of Veterinary Medicine Vienna, Vienna, Austria; ^2^University Clinic for Swine, Department for Farm Animals and Veterinary Public Health, University of Veterinary Medicine Vienna, Vienna, Austria; ^3^Chair of Food Chemistry and Molecular Sensory Science, Technische Universität München, Freising, Germany; ^4^Department of Pathobiology, Institute of Immunology, University of Veterinary Medicine Vienna, Vienna, Austria; ^5^Department of Population Health and Pathobiology, College of Veterinary Medicine, North Carolina State University (NCSU), Raleigh, NC, United States; ^6^Department of Pathobiology, Institute of Pathology and Forensic Veterinary Medicine, University of Veterinary Medicine Vienna, Vienna, Austria

**Keywords:** Cereulide, intoxication, within host translocation, *Bacillus cereus*, food poisoning, porcine (pig) model

## Abstract

*Bacillus cereus* is a gram-positive pathogen mainly known to evoke two types of foodborne poisonings. The diarrheal syndrome is caused by enterotoxins produced during growth in the intestine. In contrast, the emetic type is caused by the dodecadepsipeptide cereulide pre-formed in food. Usually, both diseases are self-limiting but occasionally more severe forms, including fatal ones, are reported. Since the mechanisms of cereulide toxin uptake and translocation within the body as well as the mechanism of its toxic action are still unknown, we used a porcine model to investigate the uptake, routes of excretion and distribution of cereulide within the host. Pigs were orally challenged with cereulide using single doses of 10–150 μg cereulide kg^-1^ body weight to study acute effects or using daily doses of 10 μg cereulide kg^-1^ body weight administered for 7 days to investigate effects of longtime, chronic exposure. Our study showed that part of cereulide ingested with food is rapidly excreted with feces while part of the cereulide toxin is absorbed, passes through membranes and is distributed within the body. Results from the chronic trial indicate bioaccumulation of cereulide in certain tissues and organs, such as kidney, liver, muscles and fat tissues. Beside its detection in various tissues and organs, our study also demonstrated that cereulide is able to cross the blood–brain–barrier, which may partially explain the cerebral effects reported from human intoxication cases. The neurobehavioral symptoms, such as seizures and lethargy, observed in our porcine model resemble those reported from human food borne intoxications. The rapid onset of these symptoms indicates direct effects of cereulide on the central nervous system (CNS), which warrant further research. The porcine model presented here might be useful to study the specific neurobiological effect in detail. Furthermore, our study revealed that typical diagnostic specimens used in human medicine, such as blood samples and urine, are not suitable for diagnostics of food borne cereulide intoxications. Instead, screening of fecal samples by SIDA-LC-MS may represent a simple and non-invasive method for detection of cereulide intoxications in clinical settings as well as in foodborne outbreak situations.

## Introduction

The reported incidence of foodborne outbreaks caused by Bacillus toxins is steadily increasing worldwide throughout the last decade. For instance, in the European Union about 600–700 confirmed cases of foodborne outbreaks linked to *B. cereus toxins* are annually reported (Messelhäußer and Ehling-Schulz, 2018). Due to the rise of reports on severe intoxications, the *Bacillus cereus* toxin cereulide is of special concern. Cereulide is a small dodecadepsipeptide toxin that evokes emesis a short time after ingestion of contaminated food (Agata et al., 1995). Usally the disease is self limiting but severe clinical cases, including fatal ones, have been described (Mahler et al., 1997; Dierick et al., 2005; Posfay-Barbe et al., 2008; Ichikawa et al., 2010; Shiota et al., 2010; Naranjo et al., 2011; Tschiedel et al., 2015). Cereulide is produced by a genetically closely related subgroup of *B. cereus* (Ehling-Schulz et al., 2005a). Like other cyclic depsipeptides, such as valinomycin, cereulide is produced enzymatically by large multifunctional protein complexes, so called non-ribosomal peptide synthetases (NRPS) (Ehling-Schulz et al., 2005b). Cereulide is able to complex monovalent cations, preferably potassium, and its highly hydrophobic character allows cereulide to diffuse through cell membranes (Mikkola et al., 1999). These two features make it an optimal ionophore, which can destroy the electrochemical gradient of membranes. For instance, it has been shown that cereulide disrupts the mitochondrial membrane potential by uncoupling the mitochondrial ATP synthesis, which results in the inhibition of the fatty acid metabolism in mammalian cells (Mahler et al., 1997; Teplova et al., 2006; Andersson et al., 2007). Furthermore, *in vitro* studies, using immortalized human cell lines, showed that already low, subemetic doses of cereulide can impair mitochondrial functionality by negatively effecting mitochondrial respiration (Decleer et al., 2018), underpinning the high biological activity of the cereulide toxin. Because of its high temperature and pH stability and its resistance to enzymatic cleavage, cereulide will neither be destroyed by food production and processing nor through the gastro-intestinal passage after consumption of contaminated food (Ehling-Schulz et al., 2004; Rajkovic et al., 2008).

So far, most case reports were associated with ingestion of contaminated rice or pasta dishes and demonstrated a wide range of disease types, including acute liver failure, rhabdomyolysis and encephalopathy. Although considerable progress has been made in the molecular and biochemical characterization of cereulide toxin synthesis, information on translocation of this highly potent toxin within the host and knowledge of the exact mechanisms of its action is rather limited (Ehling-Schulz et al., 2015). Until recently, accurate methods for toxin quantitation were missing, therefore ingested cereulide amounts of patients suffering from severe intoxication could not be reconstructed. Thus, the actual toxic dose in humans is hitherto unknown. From animal experiments in rhesus monkeys and *Suncus murinus*, a minimal emetic dose of 10–30 μg cereulide kg^-1^ body weight has been estimated (Agata et al., 1995; Shinagawa et al., 1995). Furthermore, due to the lack of information on cereulide absorption and distribution within the host as well as the lack of information on excretion routes, it is currently unknown, which clinical specimens are suited best for toxin verification. However, such information would be of utmost importance for effective and targeted intervention therapy in case of severe cereulide intoxications. Only recently, the first methods allowing an accurate quantitation of cereulide in complex matrices have been described. Based on the principle of a stable isotope dilution assay – mass spectrometry (SIDA-MS) (Bauer et al., 2010; Stark et al., 2013), an ISO method (EN/ISO/18465) has been established, allowing the quantitative detection of cereulide in complex matrices, such as foods and clinical specimens. It is expected that the EN/ISO/18465, published in January 2017, will facilitate obtaining a general overview on natural concentrations and prevalence of cereulide toxin in foods in the upcoming years. However, for gaining insights into cereulide absorption and tissue distribution within the body as well as routes of toxin excretion, *in vivo* studies in animal models are required. Due to many anatomical and physiological similarities (including the digestive and the cardiovascular system) to humans, pigs represent a suitable model for such studies (for review see Walters et al., 2011; Clouard et al., 2012). Furthermore, the pig represents a well characterized model to analyze toxicokinetics for other toxins structurally similar to cereulide, such as mycotoxins (Haschek et al., 1992; Fodor et al., 2006; Dilkin et al., 2010). In our current work, we used the pig model for deciphering the routes of cereulide translocation within the body and the routes of cereulide excretion after oral exposure. Thereby we aimed (i) to identify organs and tissues, directly affected by the cereulide toxin and (ii) to identify clinical specimens suitable for rapid and reliably cereulide diagnostics.

## Materials and Methods

This study was carried out in accordance with the recommendations of the Austrian Animal Welfare and Experiments laws, Austrian governmental ethic committees. The experimental procedures were approved by internal and Austrian governmental ethic committees (GZ 68.205/0221-II/3b/2010, GZ 68.205/0245-II/3b/201). During the whole experiment animals were under constant supervision of a veterinarian.

### Animals and Housing

In each experiment five Large White piglets, weighing 10–15 kg, were used. All piglets were derived from one single farm in Austria. The animals were placed in metabolic cages 3 days before start of the experiment for acclimatization. Water *ad libitum* and feed (Ferkelkorn, Garant Tiernahrung, Pöchlarn, Austria) were available before and during the whole experiment.

### Experimental Design and Implementation

A total of four experiments, each including four treated animals and one control pig, was performed to investigate the action of cereulide on piglets after oral intake of cereulide. Pigs were either challenged orally with a single dose (acute trials) or with daily over a period of 7 days (chronic trial) (see Table 1). One hour before cereulide toxin administration blood samples were taken by puncturing the anterior *Vena cava* and piglets were weighed for calculation of the cereulide concentration (*t* = 0 h). In the intoxication experiment with 150 μg kg^-1^ blood samples were taken 68 h before the experiment to reduce the distress for the animals at the start day of the experiments. Blood-chemical, immunological, and histological analyses of selected organs as well as quantification of cereulide in organs, tissues, urine and feces were carried out as detailed below.

**Table 1:** Overview of the pig intoxication experiments.

Experiment	Cereulide conc. per bodyweight	Administration	Duration	Blood samplings
1	10 μg kg^-1^	Single dose	48 h	0, 24, 48 h
2	30 μg kg^-1^	Single dose	48 h	0, 24, 48 h
3	150 μg kg^-1^	Single dose	48 h	0, 8, 24, 48 h
4	10 μg kg^-1^	Daily	7 days	0, 3, 7 days

### Production of the Cereulide Toxin and the Isotope Labeled Internal Standard

Cereulide, used for intoxication experiments, and ^13^C_6_-cereulide, used as internal standard for cereulide quantitation by LC MS/MS, was biosynthetically produced in high purities (>98%) as described previously (Bauer et al., 2010).

### Dietary Exposure

On body weight calculated amounts of cereulide, dissolved in ethanol, were mixed with milk (UHT milk; 3.5% fat), and fed to four piglets. Control piglets were fed with milk supplemented with similar ethanol concentrations as the experimental piglets. The experiment started after the uptake of the whole milk cereulide mixture. The milk was checked for bacterial contamination prior to the experiment.

### Clinical Parameters and Sampling

After oral administration of cereulide, piglets were monitored for unusual behavior and examined physically with special respect on rectal body temperature as well as pulse and respiration rate, every 2–3 h. Blood samples were taken from the *Vena cava cranialis*. Urine and feces were collected during the whole experiment and stored at -80°C. After the experiment pigs were anaesthetized by intramuscular injection of a bolus of ketamine (10 mg kg^-1^) and azaperone (1.3 mg kg^-1^). When sedation was deep enough cerebrospinal fluid (liquor) was taken by lumbar puncture. Finally, pigs were euthanized by intracardial injection of T61^®^. Animals were then necropsied and parts of the following organs, tissues or body fluids were taken for SIDA-MS analysis: liver, lung, heart, kidney, brain, spleen, small and large intestine, lymph nodes, gastric as well as intestinal contents, skeletal muscle, and abdominal and subcutaneous fat. Samples of liver, lung, heart, kidney, brain, spleen, small, and large intestine were collected for histological analysis.

### Blood Analysis

One milliliter EDTA-stabilized blood and 3 mL heparin-stabilized blood (worked up to serum) were screened for erythrocyte number, hemoglobin, hematocrit, mean cellular volume (MCV), mean cellular hemoglobin (MCH), mean cellular hemoglobin concentration (MCHC), leukocyte number, mean peroxidase index (MPXI), numbers of monocytes, lymphocytes, eosinophils, basophils, lymphoblasts, juvenile and segmented neutrophils, as well as glucose, urea, creatinine, total protein, aspartate amino transferase (AST), alanine amino transferase (ALT), gamma lactate dehydrogenase (GLDH), gamma glutamyl transferase (GGT), bile acid, lipase, creatine kinase, sodium, potassium, chloride, calcium, and phosphorus at the central diagnostic unit of the University of Veterinary Medicine Vienna.

Peripheral blood mononuclear cells (PBMC) were isolated from heparin-stabilized blood samples (5–8 mL) using Ficoll-Hypaque (PAA, Austria) density centrifugation as described elsewhere (Saalmüller et al., 1987). For flow cytometric (FCM) analysis PMBCs were labeled with monoclonal antibodies as indicated in Supplementary Table S1 and stained with the respective isotype-specific conjugates. For analysis of regulatory T cells, cells were fixed and permeabilized by using Foxp3 Staining Buffer Set (eBioscience) in accordance with the manufacturer‘s instructions or for B cells by a FormSap-F&P permeabilization and fixation protocol as described previously (Gerner et al., 2008; Käser et al., 2008). Samples were analyzed immediately after the final washing steps. Flow cytometric analyses were performed on a FACSCantoII (BD Bioscience). 50,000 events were collected for each sample. Data were analyzed using FACSDiva software 6.1.3 (BD Bioscience) and FlowJo software 7.6.

### Cereulide Quantification by Stable Isotope Dilution Assay Mass Spectrometry (SIDA-MS)

Collected blood samples, urine, feces, tissue and organs were freeze dried and stored at -80°C until analysis. Before extraction, liquid nitrogen was added to frozen samples and they were crushed to a powder using a mortar. Ten microgram ^13^C_6_-cereulide (for organs and feces) or 5 μg ^13^C_6_-cereulide (for urine and blood) was added and samples were incubated on a rocking table for 2 h. Samples were solved in ethanol (volume depending on weight 10–40 mL), incubated 14 h, and the supernatants were collected by centrifugation (RT, 8500 g, 10 min). For pre-purification the ethanolic extract was 1:5 diluted with water and applied onto the top of a methanol activated C18-SPE cartridge (6 mL; 1 g; Strata C18-E; Phenomenex, Aschaffenburg, Germany). The cartridge was washed with 6 mL water followed by methanol/water (4 mL; 70/30, v/v) and target compounds were eluted with ethanol (2.5 mL). Finally, samples were membrane filtrated (0.2 μm; PTFE membrane; Phenomenex, Aschaffenburg, Germany) and analyzed by means of LC-MS/MS as described previously (Bauer et al., 2010). In brief, LC-MS/MS analysis was performed using an Agilent 1200 HPLC-system connected to the API 4000QTrap LC-MS/MS (Applied Biosystems, Darmstadt, Germany) running in the positive electrospray ionization (ESI^+^, +5500 V) mode. Zero grade air served as nebulizer gas (45 psi) and turbo gas (425°C) for solvent drying (55 psi), nitrogen was used as curtain (20 psi) and collision gas (8.7 × 10^-7^ psi), respectively. Both quadrapoles were set at unit resolution. By means of the multiple reaction monitoring (MRM) mode, cereulide (*m/z* 1170.9→172.3) and ^13^C_6_-cereulide (*m/z* 1176.9→173.3) were analyzed using the mass transitions (given in brackets) monitored for a duration of 55 ms. In addition, three further mass transitions of the singly charged pseudomolecular ions ([M + H]^+^) and to the corresponding daughter fragment ions and of the corresponding ammonium ions ([M + NH_4_]^+^) to the same daughter fragment ions were recorded.

## Results

### Clinical Signs

All piglets treated with a single oral dose of 10 μg cereulide kg^-1^ body weight exhibited a transient depressive behavior for 4.5–6 h (9 h in one piglet). Additionally, recurrent seizures in lateral recumbency involving all four extremities lasting about 2–3 s were observed in half of the treated animals for about 20 min. Time periods of these neurological symptoms alternated with phases of typical vivid behavior characterized by uptake of feed or playing. One animal vomited 11 h after toxin intake. An increase of the applied cereulide dose to 30 μg kg^-1^ led to an earlier appearance of symptoms such as shivering after 1 h (2/4 piglets), followed by seizures an additional hour later. The remaining two piglets displayed seizures 4–6 h after cereulide intake. All symptoms disappeared after 7–9 h. Between periods with seizures the piglets again showed almost normal behavior. One piglet, which started to shiver after 1 h and suffered from seizures starting 6 h after toxin intake, additionally exhibited grinding of teeth and an increased salivation up to 24 h after the oral administration of cereulide. Application of 150 μg cereulide kg^-1^ led to a lethargic behavior after 1.5–2 h observed for all treated piglets. First symptoms were twitching of the snouts followed by convulsions of the whole body combined with seizures. Two piglets remained in recumbency up to 2 h after appearance of the first symptoms, meanwhile exhibiting profound lethargy, refusal of feed and with seizures over a period of up to 4.5 h. One piglet displayed attacks over 4 h and up to 12 h transient depressiveness. After 6–7 h, three of the four piglets gradually recovered. Symptoms of the fourth piglet vanished more rapidly after 3.5 h, but after 24 h gasping respiration, foam in the mouth and strong grinding of teeth were observed. The latter symptoms disappeared slowly within 8–10 h. To analyze the chronic toxicity, cereulide was administered daily in a concentration of 10 μg kg^-1^ for a period of 7 days. Similar to the acute toxicity experiment with 10 μg cereulide kg^-1^, all piglets developed a transient depressiveness after 2–6 h. Seizures could be observed in all four pigs to varying extents after 2–4 h but disappeared after 6 h each day. Two piglets exhibited seizures on 1 day, one piglet on 2 days and one piglet exhibited, additionally to the generally observed seizures, grinding of teeth and increased salivation on 4 days. All animals displayed normal pulse and respiration rates as well as inner body temperature during the experiment, except of the time periods in which the neurobehavioral abnormalities such as seizures and convulsions appeared. Histological examinations of the liver, lung, heart, kidneys, brain, spleen, and gastrointestinal tract did not reveal any notable or specific pathological changes.

### Blood Analyses: Hematology, Clinical Chemistry, and Immunology

**FIGURE 1:**
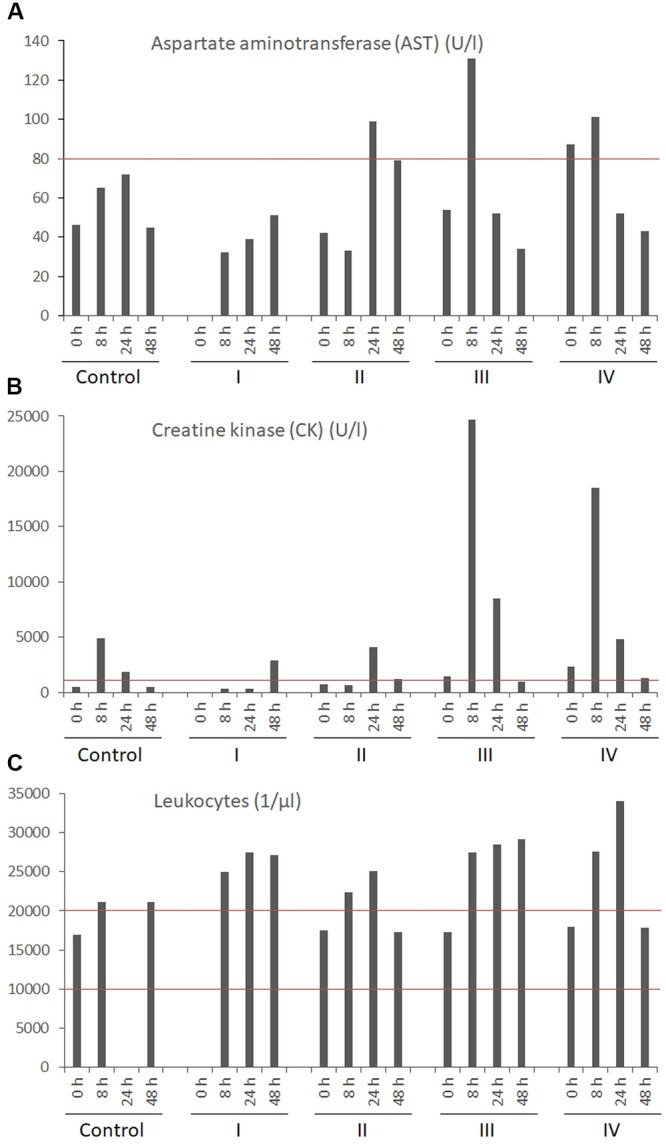
Selected blood parameters [aspartate aminotransferase **(A)**, creatine kinase **(B)**, and leukocytes **(C)**] obtained for pigs challenged with a single oral dose of 150 μg cereulide kg^-1^ body weight. Blood samples were collected 8, 24, and 48 h after dietary exposure of pigs to cereulide and blood analyses was carried our as described in Materials and Methods. Control refers to the pig receiving the same diet as the treated piglets without the cereulide toxin.

Overall no significant influence of cereulide on the blood cell composition and biochemical markers in blood samples taken before and after 8 (only 150 μg kg^-1^), 24 and 48 h were observed in cereulide treated piglets, except the levels for creatine kinase (CK) were increased in pigs challenged with 30 μg cereulide kg^-1^ and 150 μg cereulide kg^-1^, respectively. Furthermore, a transient increase of aspartate aminotransferase (AST) was observed in three out of four pigs challenged with 150 μg cereulide kg^-1^ and all animals treated with this high dose showed increased levels of leukocytes (see Figure 1). Blood parameters of samples taken in the chronic toxicity experiment were also not significantly affected by daily administration of 10 μg cereulide kg^-1^, except there was an increase of CK in all cereulide treated pigs after 3 days and elevated AST levels were found in two out of four treated pigs after 3 and 7 days, respectively (data not shown). The cell composition of PBMCs displayed no significant changes after oral administration of cereulide in comparison to control pigs (Table 2). Similar to the data of the acute toxicity experiments, the proportions of blood cell populations remained stable after daily oral administration of 10 μg cereulide kg^-1^ over a period of 7 days (data not shown).

**Table 2:** Leukocyte populations and lymphocyte subpopulations in % of total leukocytes of untreated and piglets fed with 150 μg cereulide kg^-1^ body weight.

Cell populations	Untreated piglet				Piglets fed with 150 μg cereulide kg^-^^1^ (Mean and SD)^∗^			
	0 h	8 h	24 h	48 h	0 h	8 h	24 h	48 h
B cells	26.20	25.40	27.00	18.10	28.33 ± 5.46	23.13 ± 5.52	26.05 ± 3.95	25.03 ± 5.13
B cells CD21 ^+^	14.25	15.67	13.4	9.15	15.54 ± 0.15	15.8 ± 0.31	17.7 ± 0.25	16.21 ± 0.31
Plasmacytoid DCs	0.90	1.17	0.81	1.10	0.66 ± 0.11	0.46 ± 0.09	2.19 ± 1.44	1.48 ± 0.52
Conventional DCs	0.11	0.18	0.19	0.21	0.16 ± 0.10	0.11 ± 0.07	0.11 ± 0.04	0.22 ± 0.08
Monocytes	14.10	12.90	10.80	12.00	11.53 ± 1.11	7.58 ± 4.51	10.89 ± 3.14	14.45 ± 2.51
T helper cells	16.10	15.30	11.40	17.20	14.83 ± 1.14	16.43 ± 3.31	16.70 ± 3.75	15.05 ± 3.18
CTLs	11.00	16.00	19.10	21.80	9.17 ± 3.92	17.73 ± 8.48	15.16 ± 7.09	13.65 ± 3.44
TCR γδ T cells	10.30	10.30	13.30	9.79	12.62 ± 5.91	14.05 ± 3.25	12.58 ± 3.60	10.68 ± 4.12
TCR αβ T cells	26.20	28.40	26.50	37.90	22.20 ± 4.34	31.48 ± 5.59	30.08 ± 5.32	27.60 ± 2.58
T regs	0.58	0.53	0.54	0.60	0.71 ± 0.09	0.87 ± 0.08	0.68 ± 0.19	0.70 ± 0.26
T regs CD4 ^+^	0.48	0.44	0.44	0.51	0.60 ± 0.002	0.74 ± 0.001	0.56 ± 0.007	0.57 ± 0.011
NK cells	9.44	nd	8.22	10.50	7.23 ± 3.45	9.12 ± 5.45	8.11 ± 5.00	5.83 ± 4.32

*^∗^ Mean and SD are derived from blood samples of four pigs.*

### Excretion of Cereulide

**FIGURE 2:**
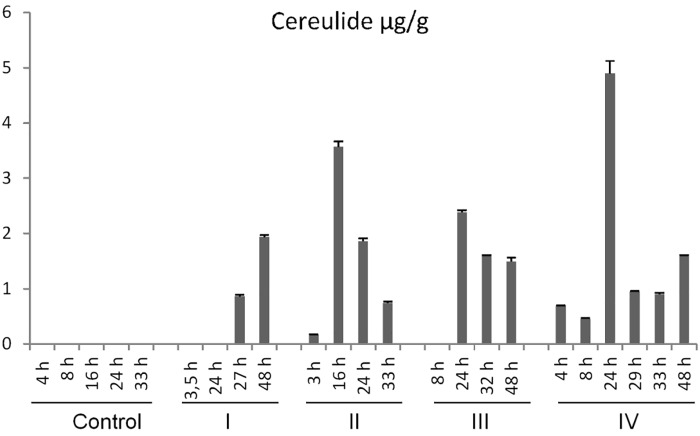
Concentrations of cereulide (μg/g) in feces of pigs challenged with a single oral dose of 150 μg cereulide kg^-1^ body weight. Fecal samples were collected after dietary exposure of pigs to cereulide for a period of 48 h. Cereulide content in the fecal samples was determined by SIDA-LC-MS, using isotope labeled ^13^C_6_-cereulide as internal standard (Bauer et al., 2010). Control refers to the pig receiving the same diet as the treated piglets without the cereulide toxin.

Urine and fecal samples collected throughout the trial were subjected to cereulide toxin analysis by SIDA-MS. Generally, high cereulide concentrations were detected in the fecal samples (up to 5 μg cereulide g^-1^ feces) while in the urine samples cereulide was only detectable at very low concentrations (up to 2 ng cereulide mL^-1^ urine) from pigs orally challenged with 150 μg cereulide kg^-1^ but not from animals challenged with lower cereulide doses (10 μg kg^-1^/30 μg kg^-1^). In pigs challenged with the high dose of 150 μg kg^-1^ toxin, cereulide was already detected in fecal samples after 3–4 h post-toxin exposure (Figure 2). Highest toxin amounts were excreted after 16–24 h, with exception of one pig that did not defecate over a period of 20 h. The overall cereulide excretion in collected fecal samples within the first 48 h after a high cereulide toxin challenge (150 μg cereulide kg^-1^) mounted up to 15% of the total quantity of the cereulide toxin administered. In case of the lower toxin challenge (30 μg cereulide kg^-1^) up to 13% of the total amount of cereulide was found in the feces within the first 48 h. For example, after 12 h 20.90 μg, after 24 h 26.80 μg and after 48 h 1.38 μg cereulide were excreted with the feces (total amount of 49.08 μg cereulide in 33.6 g feces) of a pig (13 kg) exposed to 30 μg cereulide kg^-1^ (which corresponds to a total oral intake of 390 μg cereulide), suggesting an uptake of cereulide into the body. Similar to the acute trial using a single cereulide toxin dose, no cereulide was detectable in urine samples from the chronic trail, using a daily dose of 10 μg cereulide kg^-1^ over a period of 7 days. After 2–3 days, cereulide became detectable in the fecal samples of pigs from the chronic trial and could be recovered from feces until the end of trial on day 7, in concentrations ranging from 0.02 to 0.29 μg cereulide g^-1^ feces (data not shown).

### Distribution of Cereulide Within the Host

**FIGURE 3:**
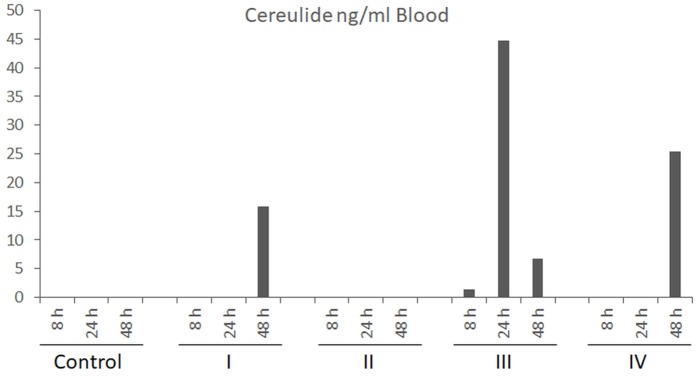
Concentrations of cereulide (ng/mL) in blood of pigs challenged with a single oral dose of 150 μg cereulide kg^-1^ body weight. Blood samples were collected 8, 24, and 48 h after dietary exposure of pigs to cereulide and concentrations of cereulide was determined as described in the legend to Figure 2. Control refers to the pig receiving the same diet as the treated piglets without the cereulide toxin.

**FIGURE 4:**
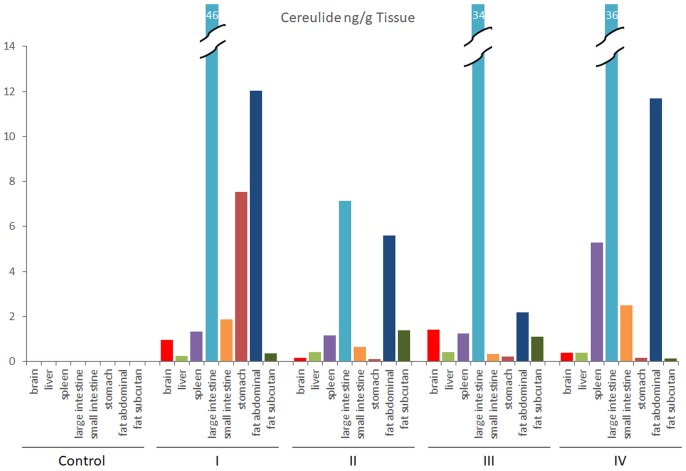
Concentrations of cereulide (ng/g) in organs of pigs challenged with a single oral dose of 150 μg cereulide kg^-1^ body weight. Animals were necropsied 48 h after dietary exposure to cereulide and concentrations of cereulide was determined in selected organs and tissues as described in the legend to Figure 2. Control refers to the pig receiving the same diet as the treated piglets without the cereulide toxin.

Blood samples, collected in parallel to the ones used for clinical-biochemical analyses, were subjected to cereulide analysis by SIDA-MS. Cereulide was detectable in three out of four pigs challenged with high dose of cereulide (150 μg kg^-1^) after 24–48 h (Figure 3) but not in the blood samples from animals receiving cereulide at lower concentrations (data not shown). Notable, the animal with the highest concentration of cereulide in the blood (45 ng cereulide mL^-1^), was the one that showed the strongest alterations in biochemical blood parameters (see Figure 1). After necropsy, samples from selected organs and tissues were taken and tested for the translocation of cereulide toxin within the host by SIDA-MS. Cereulide was detectable in various organs and tissues, although at much lower concentrations than in the feces (Table 3). Within the body, cereulide concentrations were highest in samples from the large intestine, indicating an uptake through the intestinal tract, entry into the bloodstream and distribution throughout the body. In animals challenged with the high dose (150 μg cereulide kg^-1^), cereulide was found in the content of the large intestine in concentrations up to 140 ng g^-1^ while only very low concentrations of the cereulide toxin (max. 1 ng g^-1^) were detected in the content of the small intestine. Although cereulide was detectable in the stomach, spleen, liver and brain and fat tissues of all animals challenged with the high dose, the effectiveness of translocation and uptake within the body seems to vary between individuals (Figure 4). In two out of four animals challenged with 150 μg cereulide kg^-1^ concentrations of cereulide in fat tissues of up to 12 ng cereulide g^-1^ were found while in one animal cereulide was only detectable in an amount of 2 ng cereulide g^-1^ fat tissue. Notable, the latter animal was the one with highest concentration of cereulide in blood (up to 45 ng cereulide g^-1^) as well as in the brain (1.8 ng cereulide g^-1^). It was also the animal showing the strongest alterations in biochemical blood parameters (Figure 1). Similar to the acute trials, among the organs and tissues tested, highest concentrations of cereulide were also found in the large intestine and in large intestinal contents (up to 6.8 ng g^-1^) of pigs receiving a daily oral dose of 10 μg cereulide kg^-1^ over a period of 7 days while cereulide was not detectable in the small intestine. Furthermore, cereulide was detectable in some other organs, such as spleen, kidney, liver and muscles, as well as in fat tissues, suggesting accumulation of cereulide within the body after longtime, chronic exposure (Table 3). On average the concentration of cereulide found in the content of the large intestine in pigs challenged with high cereulide dose (150 μg kg^-1^) in the acute trial were about 15-fold higher than the ones found on average in the contents of the large intestine in pigs challenged daily with low dose of cereulide (10 μg kg^-1^) in the chronic trail. By contrast, the amounts of cereulide found in muscle of the pigs from the chronic trial even exceed the amounts of cereulide found in muscles of animals challenged with high dose in the acute trial threefold, although the total amount of cereulide received by the animals in the chronic trial accounted only 47% of the amount received by the animals from the high dose group in the acute trial. Thus, it is tempting to speculate that longtime chronic expose to cereulide could lead to bioaccumulation of cereulide in certain tissues and organs. Overall the results from the feeding trials clearly indicate absorption and translocation of cereulide within the host.

**Table 3:** Cereulide concentrations (ng/g) in organs and tissue of pigs orally challenged with single doses of 30 and 150 μg cereulide kg^-1^ body weight and daily doses of 10 μg cereulide kg^-1^ body weight for 7 days.

	Single dose (acute trial)				Daily dose (chronic trial)	
	**30 μg kg^-1^**		**150 μg kg^-1^**		**10 μg kg^-1^**	
	**Range**	**Average**	**Range**	**Average**	**Range**	**Average**

Brain	–		0.15–1.41	0.73 ± 0.49	–	
Heart	–		–		–	
Kidney	–		0–0.43	0.21 ± 0.21	0.14–0.37	0.25 ± 0.12
Liver	–		0.25–0.41	0.37 ± 0.07	0.06–0.24	0.14 ± 0.08
Spleen	–		1.15–5.29	2.26 ± 1.75	0.10–0.21	0.15 ± 0.06
Lung	–		–	–	n.d.	
Lymph nodes	–		0–4.50	2.82 ± 1.71	0.01–0.14	0.07 ± 0.07
Large intestine	0–0.13	0.07 ± 0.06	7.14–45.75	30.50 ± 14.26	1.16–5.05	3.11 ± 1.95
Small intestine	–		0.34–2.49	1.34 ± 0.88	–	
Stomach	–		0.11–7.54	2.00 ± 3.20	n.d.	
Fat abdominal	–		2.17–12.02	7.88 ± 4.17	n.d.	
Fat subcutan.	–		0.35–1.37	0.74 ± 0.51	0–0.08	0.04 ± 0.04
Muscle	–		0–0.76	0.39 ± 0.32	0–2.23	1.12 ± 1.12
Content large intestine	0–0.09	0.07 ± 0.03	5.84–140.99	67.88 ± 50.80	2.04–6.78	4.41 ± 2.37
Content small intestine	n.d.		0–1.06	0.68 ± 0.53	n.d.	

*Animals were necropsied 48 h after dietary exposure to cereulide in the acute trials and 7 days after start of the experiment in the chronic trail. Concentration of cereulide was determined in organs and tissues by SIDA-LC-MS, using isotope labeled ^13^C_6_-cereulide as internal standard (Bauer et al., 2010).*

## Discussion

### Pig as Model for Foodborne Cereulide Intoxication

Although cereulide is well known for its emetic activity and hepatocytoxic potential (Ehling-Schulz et al., 2004), case reports from severe and fatal human cereulide intoxications (Dierick et al., 2005; Ichikawa et al., 2010; Shiota et al., 2010; Naranjo et al., 2011; Tschiedel et al., 2015) indicate additional, hitherto unknown, targets of cereulide inside the human body. Its lipophilic character may allow cereulide to pass through membranes, enter the bloodstream and reach different organs throughout the whole body, thereby affecting different metabolic functions. Weaning piglets were therefore used as a model host to study the effects of cereulide after oral administration and to investigate the distribution of cereulide within the body. Since the exact emetic dose of cereulide is not known in humans, we used cereulide concentrations ranging from 10 to 150 μg cereulide kg^-1^ body weight in our dose finding study. Dose selection was based on amounts of cereulide deduced from scarce reports of recent foodborne outbreaks providing information on cereulide amounts. Unfortunately, details on patients, such as body weight and amount of food consumed, are usually lacking in these reports. Based on the data available, cereulide amounts implicated in these outbreaks can be estimated to range from 80 to 150 μg cereulide kg^-1^ body weight (Naranjo et al., 2011; Delbrassinne et al., 2015; Marxen et al., 2015). It can therefore be assumed that the doses used in our porcine model are generally matching the ones reported from human outbreaks. The observed symptoms in the piglets occurred within 1.5–3 h and were self-limiting. After 7–9 h the symptoms ceased, which parallels the time period of symptoms reported from human outbreaks (Ehling-Schulz et al., 2004). In contrast to humans, emesis was only rarely observed in the pigs, rendering it likely that the mechanism of emesis induction in pigs is slightly different or that the corresponding receptors triggering emesis in pigs are not as sensitive as in humans. Today the mechanism that evokes emesis in humans in response to cereulide is unclear but based on the results from 5-HT_3_ receptor blocking experiments in *S. murinus*, it is likely that this receptor might also play an important role in emesis caused by cereulide in humans (Agata et al., 1995). It is known that pigs also possess a 5-HT_3_ receptor and the emesis provoking mechanism is thought to be similar to the one of humans (Szelenyi et al., 1994; Grelot et al., 1996; Girod et al., 2000; Girod et al., 2002). However, results of our pig feeding experiments and the observations from human intoxication cases suggest additional targets of cereulide. Similar to reports from severe human intoxications (Dierick et al., 2005; Posfay-Barbe et al., 2008; Ichikawa et al., 2010; Shiota et al., 2010), the pigs showed lethargic behavior, respiratory distress and seizures, fostering the hypothesis that pigs and humans possess common targets for cereulide toxin action. For instance, the first symptoms from a severe case report from a 11 years old boy resembled the results of the pig feeding experiments: the affected boy showed drowsiness, abdominal cramps and convulsions after ingestion of cereulide contaminated food and an acute encephalopathy was diagnosed (Ichikawa et al., 2010), indicating the central nervous system (CNS) as a major target for cereulide.

### Excretion and Translocation of Cereulide Within the Host: Implications for Diagnostics

Due to the lipophilic character of cereulide (log Kow 5.96) (Andersson et al., 2007), it could be assumed that part of the ingested cereulide adheres to digested food and is rapidly excreted via feces. Indeed, as shown in our porcine model, within the first 48 h 10–15% of the cereulide toxin orally administered is shed with feces. Cereulide was already detected in feces a few hours after toxin administration, which allows an early, non-invasive identification of cereulide intoxications. Even in case of the low dose of cereulide used in the chronic trial (10 μg cereulide kg^-1^) cereulide could be identified after 2–3 days and was quantitatively detectable in feces until the end of the trial using SIDA-LC-MS (Bauer et al., 2010). However, our pig feeding study also revealed that the current standard clinical procedures to test for intoxication, which cover only specimens such as serum and urine, are not suitable for diagnosis of cereulide and bear the risk of false negative results. Cereulide was only detectable in very low concentrations in urine and in low concentrations in blood samples in part of the pigs orally challenged with the high dose (150 μg cereulide kg^-1^) but neither in urine nor in blood samples of the ones treated with lower doses. These results from our porcine model are in line with findings from severe human cases, reporting on high amounts of cereulide in stool but rather low amounts of cereulide toxin in urine and rapid clearance of cereulide from serum (Shiota et al., 2010). A recent study of toxicokinetics of cereulide in plasma of rabbits revealed an elimination half-life of 10.8 ± 9.1 h for cereulide after its direct intravenous injection (Cui et al., 2016). Thus, in case of clinical diagnostics of suspected cases of cereulide intoxications, it is recommended to include fecal samples in the analytical pipeline. Furthermore, as shown in our current study, SIDA-LC-MS (Bauer et al., 2010; Stark et al., 2013) allows accurate quantitation of cereulide in feces, rendering feces analyses by SIDA-LC-MS a suitable method for rapid, non-invasive screening of large cohorts in frame of investigations of foodborne outbreaks, suspected to be cause by cereulide.

Similar to a fatal human case where high amounts of cereulide have been detected in the intestine content of a 1 year old boy at autopsy (Shiota et al., 2010), we detected the highest amounts of cereulide in the content of the large intestine and the tissue samples from the large intestine of the treated piglets. Considerable amounts of cereulide were also found in the abdominal fat of piglets challenged with the high dose of cereulide, suggesting absorption, accumulation and reside of cereulide in body fat, which could be explained by the lipophilic character of cereulide. Low amounts of cereulide were also detected in organs not involved in food passage processes, such as heart, muscles, and brain, indicating the ability of cereulide not only to enter the bloodstream but also to break the blood-brain-barrier (BBB) and exert its action directly on the brain. However, the amount of cereulide found in organs and fat tissues was highly variable among the individual piglets. In three out of four animals challenged with the high toxin dose, the proportion of cereulide found in the abdominal fat samples was considerable high while the proportion of cereulide in the abdominal fat of the fourth animal was rather low. The animal with the lowest proportion of cereulide in abdominal fat tissues was the one with the highest cereulide levels in blood. Cereulide was detectable in blood samples of this pig already after 8 h, reaching a maximum of 45 ng cereulide mL^-1^ after 24 h. The latter pig was also the one showing the strongest alterations in biochemical blood parameters and the highest amount of cereulide (1.4 ng cereulide g^-1^) in the brain. The observed rapid transient upregulation of CK and AST in the pigs is in line with reports from blood biochemical analyses of severe and fatal human cases (Dierick et al., 2005; Posfay-Barbe et al., 2008; Shiota et al., 2010; Tschiedel et al., 2015). The detection of cereulide in brain samples shows that cereulide can cross the BBB, which might explain the observed brain edema in a case of acute encephalopathy mimicking the Rey syndrome (Ichikawa et al., 2010).

Notable, after 1 week of daily oral challenge of the piglets with the low dose, accumulation of cereulide was observed in fat tissue as well as in some organs, including muscles. These results foster the hypothesis that in case of chronic exposure at low dose bioaccumulation of cereulide in specific human tissues and organs can occur, which may increase the potential health hazard due to modulating effects of even low concentrations of cereulide on cellular functions (Decleer et al., 2018). However, because of experimental reasons we administered food spiked with purified cereulide to the piglets. In natural intoxication scenarios it must be expected that the cereulide contaminated food also contains bacterial cell debris, which may influence translocation of cereulide within the body.

Low concentrations of cereulide can cause dysfunction in beta-cell lines (Vangoitsenhoven et al., 2014) and have been reported to be detrimental toward isolated porcine pancreatic Langerhans islets (Virtanen et al., 2008). Using Min6 cells and whole mouse pancreatic islets as model, it could be shown that low concentrations of cereulide (0.25–0.5 ng/mL) are directly effecting the insulin secretory machinery (Vangoitsenhoven et al., 2014). Based on their *in vitro* findings the authors hypothesized that cereulide may contribute to the rise of prevalence of diabetes observed worldwide, as a result of the increased consumption of rice, which is frequently contaminated with *B. cereus*. Further *in vivo* studies will be needed to elucidate the importance and potential risks associated with longtime dietary exposure to cereulide at low concentrations.

### Cereulide, a Potent Neurotoxin?

Remarkably, all cereulide treated pigs in this study exhibited neurobehavioral abnormalities, which are indicative for an involvement of the CNS. Manifestation and duration of symptoms increased with rising cereulide concentrations. In low concentrations cereulide was detected in the brain, indicating an uptake of the toxin in the bloodstream, thereby breaking the BBB and reaching the brain, where it may influence potassium concentrations due to its ionophoretic properties. Animal epilepsy models showed that one cause for seizures is aberrations in the potassium regulation in the brain (Frohlich et al., 2008). Due to its lipophilic character cereulide might be able to cross the BBB by transmembrane diffusion (Oldendorf, 1974), possibly leading to a disturbance of the potassium content of the cerebrospinal fluid. Potassium is normally prevented by the BBB from reaching the cerebrospinal fluid (Durand et al., 2010). The ionophoretic properties of cereulide could affect that barrier function and potassium may be transported from blood (physiologically 5 mM K^+^) to the cerebrospinal fluid (2.9 mM K^+^). As shown previously, high potassium concentrations are involved in the generation of synaptic seizures (Durand et al., 2010), which might explain the seizures observed in response to cereulide. Some of the symptoms, such as lethargy, seizures, and convulsions, observed after oral administration of cereulide to pigs resemble the symptoms reported from human cases (Mahler et al., 1997; Dierick et al., 2005; Posfay-Barbe et al., 2008; Ichikawa et al., 2010; Shiota et al., 2010; Naranjo et al., 2011). Lethargy was observed in almost all patients a few hours after ingestion and seizures/convulsions were noticed up to 6 h after ingestion. The reported mild intelligence impairments of a boy suffering from a severe foodborne intoxication caused by cereulide might, at least partially, be explained by direct action of the toxin on the brain in a yet to explore manner.

## Conclusion

In conclusion, this is, to our knowledge, the first *in vivo* cereulide intoxication study, using the pig as model. It could be shown that the highly stable toxin cereulide is partly absorbed within the body and could be detected in some organs, including the brain. Thus, our study demonstrated that cereulide is able to cross the BBB and could exert direct actions on the brain, which may partially explain the cerebral effects reported from severe cereulide intoxication cases. Additionally, it was demonstrated that typical diagnostic specimens used in human medicine, such as blood samples and urine, are not suitable for diagnostics of cereulide intoxications. Since considerable amount of cereulide is excreted via feces, screening of fecal samples may represent a simple and non-invasive method for detection of cereulide intoxications in clinical settings and in foodborne outbreak situations. Neurobehavioral symptoms such as seizures and lethargy, which are also described in severe human case reports, were observed shortly after ingestion of cereulide, indicating direct effects on the CNS. This observed specific neurobiological effect has to be analyzed in detail. Thus, the impact of cereulide on neurons or its influence on the cerebral potassium concentrations remains elusive and warrants further investigations.

## Author Contributions

ME-S and TB conceived and design the study and wrote the manuscript. TB, WS, CK performed the animal experiments. WS coordinated the animal experiments. RB carried out histological analyses. TB, WS, TK, TS, AS, TH, and ME-S carried out data analyses and interpretation. ME-S acted as overall study coordinator. All authors revised the manuscript.

## Conflict of Interest Statement

The authors declare that the research was conducted in the absence of any commercial or financial relationships that could be construed as a potential conflict of interest.
