# Supplementary material for: First Insights Into Within Host Translocation of the Bacillus cereus Toxin Cereulide Using a Porcine Model
Source: Front Microbiol. 2018 Nov 7;9:2652. doi: 10.3389/fmicb.2018.02652 (PMC6234764; doi:10.3389/fmicb.2018.02652)
Supplement: Supplementary file 1 [file Table_1.pdf]

Table S1: Scheme of PBMC labeling for FCM (for details of labeling see materials and methods)

| Staining of Cell populations | First antibodies | Source | Second antibodies | Source | Fix /Perm | Intracell. Antigen staining | Source |
|---|---|---|---|---|---|---|---|
| Negative control | - | | - | | - | - | |
| Isotype control | Isotyp control IgG2b<br>Isotyp control IgG<br>Isotyp control IgG2a | 1<br>1<br>1 | Anti-IgG2b-Alexa488<br>Anti-IgG1-RPE<br>Anti-IgG2a-Alexa647 | 2<br>3<br>2 | - | - | |
| T helper cells<br>NK cells | Anti-CD4 (74-12-4; IgG2b)<br>Anti-CD3 (PPT3; IgG1)<br>Anti-CD8α (11/295/33; IgG2a) | 4<br>5<br>6 | Anti-IgG2b-Alexa488<br>Anti-IgG1-RPE<br>Anti-IgG2a-Alexa647 | 2<br>3<br>2 | - | - | |
| T-helper<br>Cytotoxic T lymphocytes | Anti-CD4 (74-12-4; IgG2b)<br>Anti-CD45RC (3a56; IgG1)<br>Anti-CD8ß (PG164A; IgG2a) | 4<br>7<br>8 | Anti-IgG2b-Alexa488<br>Anti-IgG1-RPE<br>Anti-IgG2a-Alexa647 | 2<br>3<br>2 | - | - | |
| γδ T cells | Anti-TcR-gd (PPT16; IgG2b)<br>Anti-CD3 (PPT3; IgG1)<br>Anti-CD8α (11/295/33; IgG2a) | 8<br>5<br>6 | Anti-IgG2b-Alexa488<br>Anti-IgG1-RPE<br>Anti-IgG2a-Alexa647 | 2<br>3<br>2 | - | - | |
| Dendritic cells monocytes | Anti-CD4 (74-12-4; IgG2b)<br>Anti-SWC3/CD172a (74-22-15; IgG1)<br>Anti-CD14 (TYK4; IgG2a) | 4<br>8<br>9 | Anti-IgG2b-Alexa488<br>Anti-IgG1-RPE<br>Anti-IgG2a-Alexa647 | 2<br>3<br>2 | - | - | |
| Regulatory T cells | Anti-CD4 (74-12-4; IgG2b)<br>Anti-CD25(3B2; IgG1) | 4<br>10 | Anti-IgG2b-Alexa488<br>Anti-IgG1-RPE | 2<br>3 | Yes Ebioscience Fix Perm | Anti-Foxp3 (FJK-16s; IgG2a)-Alexa647 | 12 |
| B-cells | Anti-CD21-APC (B-ly4; IgG1) | 11 | - | | Yes saponin | Anti-CD79 (HM57; IgG1)-PE | 13 |

**1** Dianova, Hamburg, DE **2** Invitrogen, Carlsbad **3** Southern Biotech, Birmingham, UK **4** (Pescovitz *et al.*, 1984) **5** (Yang *et al.*, 1996) **6** (Jonjic & Koszinowski, 1984) **7** (Zuckermann *et al.*, 1994a; Zuckermann *et al.*, 1994b) **8** Institute for Clinical Immunology, University of Veterinary Medicine Vienna **9** Serotec Raleigh, NC, **10** (Bailey *et al.*, 1992) **11** BD Bioscience, San Jose, CA **12** eBioscience. San Diego, CA **13** Dako Glostrup, DK


**Pescovitz, M. D., Lunney, J. K. & Sachs, D. H. (1984).** Preparation and characterization of monoclonal antibodies reactive with porcine PBL. *Journal of immunology* **133**, 368-375.

**Yang, H., Oura, C. A., Kirkham, P. A. & Parkhouse, R. M. (1996).** Preparation of monoclonal anti-porcine CD3 antibodies and preliminary characterization of porcine T lymphocytes. *Immunology* **88**, 577-585.

**Jonjic, S. & Koszinowski, U. H. (1984).** Monoclonal antibodies reactive with swine lymphocytes. I. Antibodies to membrane structures that define the cytolytic T lymphocyte subset in the swine. *Journal of immunology* **133**, 647-652

**Zuckermann, F. A., Binns, R. M., Husmann, R., Yang, H., Carr, M. M., Kim, Y. B., Davis, W. C., Misfeldt, M. & Lunney, J. K. (1994a).** Analyses of monoclonal antibodies reactive with porcine CD44 and CD45. *Veterinary immunology and immunopathology* **43**, 293-305.

**Zuckermann, F. A., Schabacker, D. & Binns, R. M. (1994b).** Biochemical analysis of molecules reactive with monoclonal antibodies specific for porcine CD45. *Veterinary immunology and immunopathology* **43**, 307-313.

**Bailey, M., Stevens, K., Bland, P. W. & Stokes, C. R. (1992).** A monoclonal antibody recognising an epitope associated with pig interleukin-2 receptors. *Journal of immunological methods* **153**, 85-91.
